# The illusory truth effect leads to the spread of misinformation

**DOI:** 10.1016/j.cognition.2023.105421

**Published:** 2023-07

**Authors:** Valentina Vellani, Sarah Zheng, Dilay Ercelik, Tali Sharot

**Affiliations:** a Affective Brain Lab, Department of Experimental Psychology, University College London, London WC1H 0AP, UK; b Max Planck University College London Centre for Computational Psychiatry and Ageing Research, London WC1B 5EH, UK; c Department of Brain and Cognitive Sciences, Massachusetts Institute of Technology, Cambridge, MA, USA

**Keywords:** Information-sharing, Illusory truth effect, Misinformation, Repetition

## Abstract

Misinformation can negatively impact people's lives in domains ranging from health to politics. An important research goal is to understand how misinformation spreads in order to curb it. Here, we test whether and how a single repetition of misinformation fuels its spread. Over two experiments (*N* = 260) participants indicated which statements they would like to share with other participants on social media. Half of the statements were repeated and half were new. The results reveal that participants were more likely to share statements they had previously been exposed to. Importantly, the relationship between repetition and sharing was mediated by perceived accuracy. That is, repetition of misinformation biased people's judgment of accuracy and as a result fuelled the spread of misinformation. The effect was observed in the domain of health (Exp 1) and general knowledge (Exp 2), suggesting it is not tied to a specific domain.

## 1 Introduction

Engagement with misinformation online has doubled in recent years. Misinformation about COVID-19, for example, proliferated over the last year with 1.1 million articles containing misinformation about COVID-19 shared on social media (Evanega, Lynas, Adams, Smolenyak, & Insights, 2020). Such growth has concerning consequences including the increase of vaccines hesitancy, polarization, violent extremism and racism (Barreto et al., 2021; Newman, Lewandowsky, & Mayo, 2022; Rapp & Salovich, 2018; Swire-Thompson & Lazer, 2019; Tsfati et al., 2020). For instance, misleading information on how to treat COVID-19 can lead to delays in properly treating patients. To halt the spread of misinformation it is crucial to identify the mechanisms facilitating its spread. Here, we ask whether and how a single previous exposure to misinformation alters the likelihood that it will be shared.

A vast literature suggests that repeated statements are perceived as more accurate (Arkes, Boehm, & Xu, 1991; Arkes, Hackett, & Boehm, 1989; Bacon, 1979; Begg, Anas, & Farinacci, 1992; Hasher, Goldstein, & Toppino, 1977; Hawkins & Hoch, 1992; Law, Hawkins, & Craik, 1998; Roggeveen & Johar, 2002; Johar & Roggeveen, 2007; Begg, Armour, & Kerr, 1985; Begg & Armour, 1991; Brown & Nix, 1996; Doland, 1999; Gigerenzer, 1984; Hawkins, Hoch, & Meyers-Levy, 2001; Law & Hawkins, 1997; Schwartz, 1982; Fazio, Brashier, Payne, & Marsh, 2015; Swire, Ecker, & Lewandowsky, 2017; for a review Dechêne, Stahl, Hansen, & Wänke, 2010) even when statements are from non-credible sources (Begg et al., 1992; Fazio, Rand, & Pennycook, 2019; Murray, Stanley, McPhetres, Pennycook, & Seli, 2020; Pennycook, Cannon, & Rand, 2018). This phenomenon is known as “The Illusory Truth Effect” (Arkes et al., 1989; Fazio et al., 2019; Murray et al., 2020) and has been shown in domains ranging from marketing (Hawkins & Hoch, 1992; Johar & Roggeveen, 2007; Law et al., 1998; Roggeveen & Johar, 2002) to news (Murray et al., 2020; Pennycook et al., 2018). A single previous exposure to missinformation can increase perceived accuracy even when the information is inconsistent with the participant's ideology (Murray et al., 2020; Pennycook et al., 2018). Importantly, previous studies show that people tend to share more accurate information than misinformation (Epstein et al., 2021; Pennycook et al., 2021; Pennycook, McPhetres, Zhang, Lu, & Rand, 2020; Pennycook & Rand, 2022) and explicitly say they prefer to do so (Epstein et al., 2021; Pennycook et al., 2021). Other factors can, and certainly do, drive information-sharing (Chen, Pennycook, & Rand, 2021; Pennycook et al., 2021; Van Bavel et al., 2020; Pereira and Van Bavel, 2018), for example - motivation. Nonetheless, accuracy is one driving factor.

We pose that if (i) people on average tend to share information they believe is true more than information they believe is false and (ii) repetition increases perceived accuracy, then repeated information will be shared more than new information because people will believe it is accurate (for similar theoretical prediction see Van Bavel et al., 2020 and Effron & Helgason, 2022).

We test this hypothesis within the domain of health and general knowledge. Misinformation in these domains is commonplace and can have significant societal consequences. A few such examples include the belief that the earth is flat (geography), that evolution is false (science), that the holocaust never happened (history), that climate change is not men-made (science) and that COVID vaccines are dangerous (health). Generalizing across a variety of domains is important in showing the domain-generality of the effect. This is critical for both theory and practice.

Previous attempts to test similar hypothesis focused specifically on sharing of political information (Effron & Raj, 2020; Fazio, 2020). Political beliefs are known to be especially influenced by motivation, and they are strongly tied to self-identity and are polarizing. These factors will have a strong influence on information sharing, which may overshadow the effect of repetition. In other words, within the political domain the effect size of repetition on sharing may be smaller, which may explain why within this domain mixed results have been observed (Effron & Raj, 2020; Fazio, 2020).

Here, we run an information-sharing task in which participants indicated whether they would like to share health-related statements (Exp 1) and general knowledge statements (Exp 2) with other participants on social media. Half of the statements were repeated, and half were new. In addition, participants indicated whether they perceived each statement as true of false. This allowed us to test whether repetition increases belief in accuracy, even when statements are false, leading to increased sharing of misinformation.

## 2 Methods

### 2.1 Participants

Participants were recruited via Prolific Academic (https://www.prolific.co/) and were paid £7.50 per hour for their participation. The task was created using Gorilla Experiment Builder (www.gorilla.sc; Anwyl-Irvine, Massonnié, Flitton, Kirkham, & Evershed, 2020) and the JsPsych library (de Leeuw, 2015). The sample size for was determined based on previous studies on the truth effect (Dechêne et al., 2010). The study was approved by the departmental ethics committee at UCL. 162 subjects participated in Exp 1. Data from one subject was eliminated as they completed one phase of the experiment twice and data from one subject failed to save correctly. Thus, data from 160 participants was analyzed in Exp 1 (44 males, 115 females, 1 prefer not to say/other; mean age = 35.26 years ±11.05 (SD)). 102 subjects participated in Exp 2. Data from two subjects was eliminated as they completed one phase of the experiment. Thus, data from 100 subject was analyzed (53 males, 45 females, 2 other; mean age = 32.81 years ±8.99 (SD)).

A post hoc Power Analysis performed on the main effect of repetition (Partial Eta Squared: Exp1: 0.071; Exp2: 0.099, Sample size: Exp1: 160; Exp2: 100; Numerator df = 1; Number of groups = 2) on sharing intentions revealed (using G*Power) an achieved power of 0.93 for Exp 1 and 0.90 in Exp 2 (α = 0.05).

### 2.2 Procedure – Exp 1

In block 1, participants observed 30 health-related statements (selected from the Internet – see supplement material for more information) in random order, each for 6 s. Statements were randomly selected for each participant from a list of 60 (see supplement material for all statements). Half of the statements were true and half were false.

In either the second or third block (counterbalanced across participants) participants observed 60 statements (half were new, half repeated) one at a time, in a random order. For each, they indicated whether they wanted to share the statement with participants who might complete a similar task the following day. They did so using a continuous scale ranging from 1 “Not at all” to 100 “Very much”, self-paced.

In either the second block or third block (counter balanced across participants), participants indicated whether they thought each statement was true or false on a continuous scale ranging from 1 “Definitely False” to 100 “Definitely True” (Accuracy Judgment Block). Sentences were presented in a random order. This was self-paced.

In Exp 1, 77 participants completed the Accuracy Judgment Block first, and 83 completed the Information-Sharing Block first.

Attention Check: In block 1 four attention check were included. In two of these trials participants observed a statement (which was not drawn from the 60 statements list) and subsequently were presented with a list of three statements which included the one they previously saw. Their task was to indicate the previously seen statement. In the other two trials, after the presentation of the statement (which was not drawn from the 60 statements list), participants were asked to answer a question about the statement they had just seen.

In block 2 and 3, six attention check trials were inserted. Four of these trials were identical to check trials in block 1. In the other two trials, instead of indicating their sharing decision or accuracy judgment, subjects were instructed to select a specific rating (for example: *Select Definitely False*). In Exp 1, selecting one to ten was considered correct on this attention check. Participants answered correctly on 89.22% of the attention checks in Exp 1.

Between block 1 and the block 2, participants filled questions about their social-media use. After completing the study, participants filled the BES (Basic Empathy Scale).

### 2.3 Procedure – Exp 2

The task was identical to the task used in Exp 1, with three exceptions

(i) Information was ‘general knowledge’.
(ii) Participants indicated their answers on a six-point Likert scale.
(iii) The sharing scenario was hypothetical. Participants were told they had to manage a Twitter account that specialize in general knowledge. In the information-sharing block, they indicated whether they wanted to share the statement on their hypothetical Twitter account.

57 participants completed the Accuracy Judgment Block first, and 43 completed the Information-Sharing Block first. Participants answered correctly on 80.75% of the attention checks.

Most of the work on The Illusory Truth Effect has been done previously on general knowledge statements (for example: Arkes et al., 1989; Bacon, 1979; Begg et al., 1992; Brashier, Eliseev, & Marsh, 2020; Doland, 1999; Fazio et al., 2015; Hasher et al., 1977; Newman et al., 2022; Swire et al., 2017). In Exp 1, we used the same exact statements used in these previous studies (Arkes et al., 1989; Brashier et al., 2020; Fazio et al., 2015; Newman et al., 2022). General knowledge statements in Exp 1 included a broad range of facts about science (19 statements), geography (14 statements), sport (10 statements), food (7 statements), culture (5 statements), literature (3 statements) and history (2 statements) (see the Supplementary Materials).

The reason we asked participants to imagine they have an account related to health (Exp 1) or general knowledge (Exp 2) was that if we asked them to imagine their own account they may not share any statements at all, thus it would be impossible to examine the hypotheses. The reason they might not share anything at all is that people usually have accounts in which they share information from specific domains. For example, academics tend to posts about academia and science.

### 2.4 Analysis

We run two linear mixed model for each experiment, one predicting sharing decisions and the other predicting perceived accuracy. Repetition (repeated/new) and ground truth (true/misinformation) and their interactions were modelled as fixed and random effect. Random and fixed intercepts were also included.

We also run linear mixed effect models separately for true information and misinformation predicting perceived accuracy and sharing from repetition (repeated/new) only. When running the analysis on true information only, data of one subject in Exp1 (for the perceived accuracy model) and seven subjects in Exp2 (for the sharing model) caused the final Hessian matrix not to be positive definite, producing inflated degrees of freedom, due to lack of variability in their data. These subjects were thus removed from those specific analyses. Note that not removing the subjects leads to the same exact results but with inflated degrees of freedom.

To test the robustness of these results, for each subject we computed the average rating on the sharing scale and the average rating on the perceived accuracy scale, separately for repeated and new statements, and for true and false statements. A repeated measures ANOVA was conducted with repetition (new/ repeated) and ground truth (true/ misinformation) as within subject variables, and perceived accuracy as the dependent variable. The same ANOVA was conducted also with sharing ratings as the dependent variable.

We then performed a mediation analysis for each participant, using the R package Process 4.0 with 10,000 permutations, to test whether perceived accuracy mediated the effect of repetition on sharing decision. Significance of the mediation was determined by the Index of indirect effect. We could not estimate the mediation model for 1 subject in Exp 1 and 7 in Exp 2 due to non-sufficient variability in responses (that is subjects either made the exact same sharing response on all trials and/or used only two numbers on the accuracy scale). Therefore, the mediation model was estimated for 159 participants in Exp 1 and for 93 in Exp. The total effect cannot be estimated when subjects used only two numbers on the sharing scale, this was true for 6 subjects in Exp 2. Estimates were compared to 0 across participants using a *t*-test.

## 3 Results

### 3.1 Task. Exp 1

**Fig. 1:**
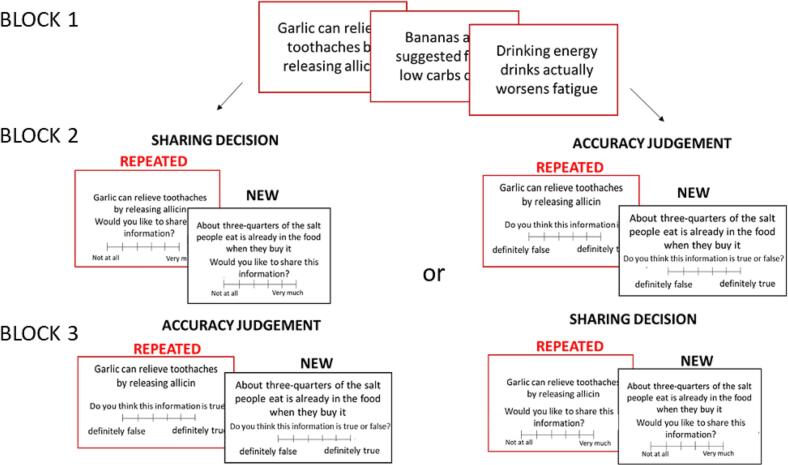
Task. In the first block, participants observed 30 health-related statements randomly selected from a list of 60. On the second or third block (counterbalanced) participants observed each of the 60 statements and indicated whether they would have liked to share the information with participants who may be conducting a similar task the following day. They replied on a continuous scale ranging from 1 “Not at all” to 100 “Very much”. In the second or third block (counterbalanced), participants indicated whether they believed each statement was true or false using a continuous scale ranging from 1 “Definitely False” to 100 “Definitely True”. Red color is used for illustrative purposes only. (For interpretation of the references to color in this figure legend, the reader is referred to the web version of this article.)

To investigate whether, and why, repeated information is shared more than new information, 160 participants performed an information-sharing task (Fig. 1). On one block of trials, they indicated whether they would like to share health-related statements (e.g., ‘For better health, one needs to remove sugar entirely from one's diet’) with participants who may be completing a similar task on the following day. Half of the statements were true and half were false (see supplementary material for full instructions).

Half of the statements (randomly assigned) were previously presented to the participants and half were new. On another block of trials, participants rated whether they believed each statement was true or false (accuracy judgment). The order of the sharing block and the accuracy judgment block was counterbalanced across individuals.

### 3.2 Repeated information is perceived as more true

**Fig. 2:**
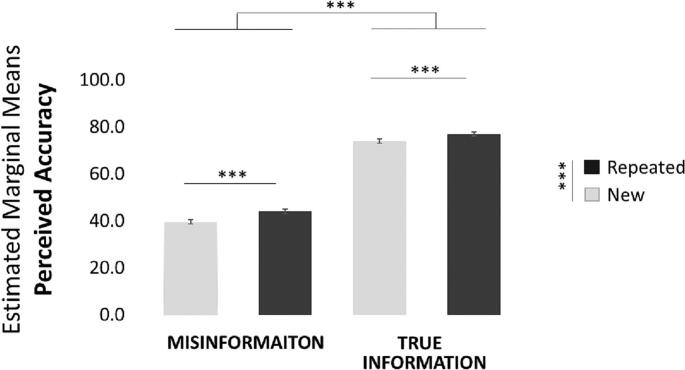
Repeated information is perceived as more accurate than new information. Plotted are the Estimated Marginal Means from the Linear Mixed Model Predicting Perceived Accuracy. Error bars indicate SEM. * *p* < 0.05, ** *p* < 0.01, *** *p* < 0.001.

We first tested for the “Illusory truth effect” - that is whether participants are more likely to perceive repeated information as true. To that end we performed a linear mixed model predicting perceived accuracy. Ground truth (true/misinformation) and repetition (repeated/new) and their interactions were modelled as fixed and random effects. Random and fixed intercepts were also included. In accordance with the literature on the “Illusory truth effect”, we found a main effect of repetition, such that repeated statements were perceived as more accurate than new statements (β = −3.12, t(315.98) = −3.85, *p* < 0.001, Fig. 2). Also, true information was perceived more accurate than misinformation (β = −33, t(251.78) = −29.88, p < 0.001). No other effects were significant. We then run a linear mixed effect model predicting perceived accuracy from repetition, separately for true information and misinformation. Random and fixed intercepts were also included. Results suggest that repetition increases perceived accuracy both for true information (β = −3.16, t(158.36) = −5.34, *p* < 0.001) and misinformation (β = −4.39, t(159.503) = −4.46, p < 0.001).

The above results are also observed when submitting average accuracy ratings for each participant into a 2 × 2 repeated measures Anova with ground truth (true/misinformation) and repetition (repeated/new) as within subject variables and perceived accuracy as the dependent variable. Once again, we found a main effect of repetition - repeated statements (M = 60.51, SD = 10.04) were rated as more true than new statements (M = 56.77, SD = 8.91; F(1,159) = 39.92, p < 0.001, η^2^ = 0.201). This was found for both true statements (Repeated: M = 76.98, SD = 8.96, New: M = 73.80, SD = 9.14, t(159) = 5.419, p < 0.001) and misinformation (Repeated: M = 44.00, SD = 14.52, New: M = 39.62 SD = 12.36, t(159) = 4.482, p < 0.001). Moreover, we found a main effect of ground truth - true information (M = 75.46, SD = 8.25) was judged as more true than misinformation (M = 41.82, SD = 12.06; F(1,159) = 1222.202, p < 0.001, η^2^ = 0.885). No other effects were significant.

### 3.3 Repeated information is shared more than new information

**Fig. 3:**
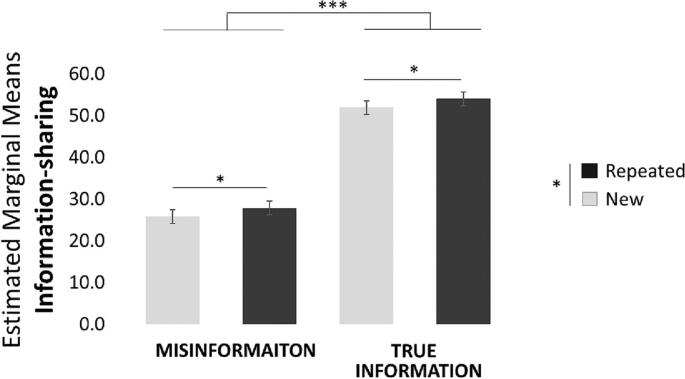
People are more likely to share information they have been previously exposed to and are also more likely to share true information. Plotted are the Estimated Marginal Means from the Linear Mixed Models Predicting Perceived Accuracy. Error bars indicate SEM. * *p* < 0.05, ** *p* < 0.01, *** *p* < 0.001.

Next, we tested whether repeated statements were shared more than new statements. A linear mixed model predicting sharing decisions was run with repetition (repeated, new), ground truth (true, misinformation) and their interactions modelled as fixed and random effect. Random and fixed intercepts were also included. The results revealed that repeated statements were shared more than new statements (β = −2.08, t(317.35) = −2.52, *p* = 0.012; Fig. 3). In addition, true information was shared more than misinformation (β = −26.20, t(229.98) = −19.60, *p* < 0.001; Fig. 3). No other effects were significant. We then run linear mixed effect models predicting sharing from repetition, separately for true information and misinformation. Random and fixed intercepts were also included. Results suggest that repetition increases sharing both for true information (β = −2.06, t(158.75) = −2.46, *p* = 0.015) and misinformation (β = −2,00, t(158.51) = −2.46, p = 0.015).

We observe the same findings when performing a 2 × 2 repeated measures ANOVA with ground truth (true/misinformation) and repetition (repeated/new) as within subject variables and sharing decision as the dependent variable. Once again, we found a main effect of repetition: people shared repeated statements (M = 41.03, SD = 20.16) more than new statement (M = 38.81, SD = 18.45; F(1,159) = 12.066, *p* = 0.001, η^2^ = 0.071). This was observed both for true statements (Repeated: M = 53.92, SD = 23.90, New: M = 51.88, SD = 22.34, t(159) = 2.437, *p* = 0.016) and misinformation (Repeated: M = 27.78, SD = 19.27, New: M = 25.78 SD = 17.38, t(159) = 2.478, *p* = 0.014). There was also a main effect of ground truth; people share true statements (M = 53.02, SD = 22.58) more than misinformation (M = 26.83, SD = 17.78; F(1,159) = 475.925, p < 0.001, η^2^ = 0.750). No other effects were significant.

### 3.4 The effect of repetition on sharing is fully mediated by perceived accuracy

**Fig. 4:**
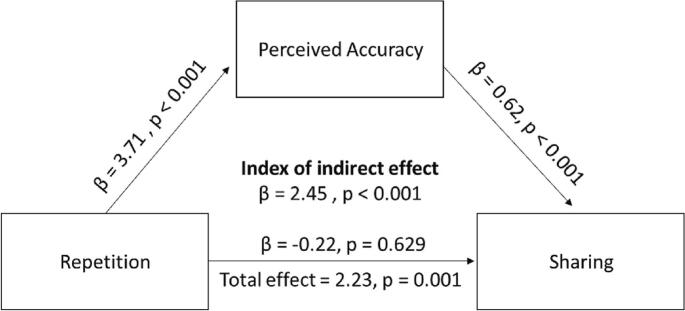
The effect of repetition on sharing is fully mediated by perceived accuracy. Repeated information is perceived as more accurate, which increases sharing of that information. The figure represents the mediation model and the Beta Coefficients obtained. *** *p* < 0.001.

So far, we found that repeated information is more likely to be shared by participants than new information. A possible underlying mechanism is that repetition boosts perceived accuracy, which in turn leads to greater sharing. To test this possibility, we performed a mediation analysis for each participant and tested the obtained estimates against zero. We found that repetition was related to greater sharing (Total effect = 2.23, SD = 8.71, t(158) = 3.235, p = 0.001, Fig. 4.) The effect of repeated exposure on information-sharing was fully mediated by perceived accuracy (Index of indirect effect = 2.45, SD = 6.42, t(158) = 4.814, *p* < 0.001). Specifically, repetition of information was associated with higher perceived accuracy (β = 3.71, SD = 9.02, t(158) = 5.188, p < 0.001) and perceived accuracy was associated with greater sharing (β = 0.62, SD = 0.32, t(158) = 24.25, p < 0.001). After accounting for perceived accuracy the relation between repetition and sharing was not significant (β = −0.22, SD = 5.64, t(158) = 0.485, *p* = 0.629).

### 3.5 Exp 2: Results generalize beyond the health domain

So far, we found that repetition leads to increased information sharing of health-related information. Next, we examined whether the results would generalize to information related to different domains. To that end, 100 participants completed the same task as in Exp 1 except that the information was ‘general knowledge’ (e.g., ‘The Cyclops is the legendary one-eyed giant in Greek mythology’). Statements included were from the following domains: science (19 statements), geography (14 statements), sport (10 statements), food (7 statements), culture (5 statements), literature (3 statements), and history (2 statements). All statements were extract from past studies on the illusory truth effect (Arkes et al., 1989; Brashier et al., 2020; Fazio et al., 2015; Newman et al., 2022).

There were two other differences between Exp 2 and Exp 1: (i) instead of being told that they would be deciding which information to share with participants that may be completing the task the next day, participants were told that they were managing a social media page about general knowledge, and they had to decide which information they would like to post. (ii) We used a 6-point Likert scale for all ratings.

**Fig. 5:**
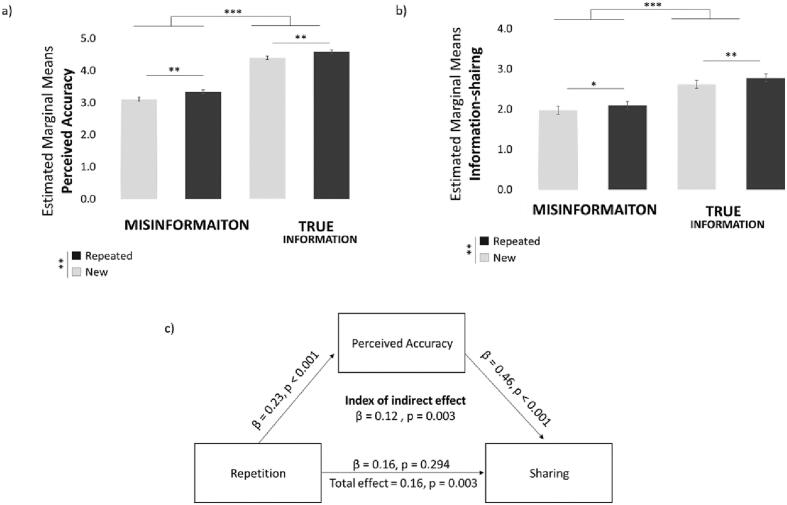
Results generalize beyond the health domain. (a) Estimated marginal means from the linear mixed models predicting perceived accuracy show that repeated information is perceived as more accurate than new information. (b) Estimated marginal means from the linear mixed models predicting sharing show that people share repeated information more than new. Error bars indicate SEM. (c) Perceived accuracy fully mediates the relationship between repetition and information-sharing. The figure represents the mediation model and the Beta Coefficients obtained. * *p* < 0.05, ** *p* < 0.01, *** *p* < 0.001.

The analysis was exactly as in Exp 1. Replicating previous results, the mixed linear model revealed that repeated statements were perceived as more accurate (β = −0.19, t (247.941) = −2.92, *p* = 0.004; Fig. 5a) than new statements. In addition, true information was perceived more accurate (β = −1.24, t (238.82) = −18.59, *p* < 0.001; Fig. 5a) than misinformation. No other effects were significant. Separate mixed linear models on true and misinformation separately showed that repetition increases perceived accuracy both for true information (β = −0.19, t(98.59) = −2.94, p = 0.004) and misinformation (β = −0.24, t(99.208) = −3.58, *p* = 0.001).

As in Exp1, results of the ANOVA were in accord with the mixed model - repeated statements (M = 3.97, SD = 0.57) were rated as more true than new statements (M = 3.75, SD = 0.37; F(1,99) = 15.949 *p* < 0.001, η^2^ = 0.139). This was observed for both true statements (Repeated: M = 4.59, SD = 0.58, New: M = 4.40, SD = 0.46, t(99) = 2.842, *p* = 0.005) and misinformation (Repeated: M = 3.34, SD = 0.72, New: M = 3.11 SD = 0.52, t(99) = 3.498, p = 0.001). Moreover, true information (M = 4.49, SD = 0.40) was judged as more true than misinformation (M = 3.22, SD = 0.53; F(1,99) = 541.264, p < 0.001, η^2^ = 0.845). No other effects were significant.

As for sharing, again the mixed model revealed that repeated statements were shared more than new statements (β = −0.16, t (267.27) = −2.91, p = 0.004; Fig. 5b). In addition, true information was shared more than misinformation (β = −0.68, t (154.46) = −8.99, p < 0.001; Fig. 5b). No other effects were significant. Linear Mixed effect model predicting sharing from repetition, separately for true information and misinformation show that repetition increased sharing both for true information (β = −0.15, t(91.92) = −2.83, *p* = 0.006) and misinformation (β = −0.12, t(99.44) = −2.26, *p* = 0.026).

A repeated measures ANOVA confirmed the results. People shared repeated statements (M = 2.43, SD = 0.95) more than new statements (M = 2.30, SD = 0.86; F(1,99) = 10.886, p = 0.001, η^2^ = 0.099). This was observed for both true statements (Repeated: M = 2.77, SD = 1.16, New: M = 2.62, SD = 1.06, t(99) = 3.139, *p* = 0.002) and misinformation (Repeated: M = 2.10, SD = 0.87, New: M = 1.97 SD = 0.78, t(99) = 2.233, *p* = 0.028).They also shared true statements (M = 2.70, SD = 1.08) more than misinformation (M = 2.03, SD = 0.78; F(1,99) = 94.482, p < 0.001, η^2^ = 0.488). No other effects were significant.

As in Exp 1, repetition was related to increase sharing (Total effect = 0.16, SD = 0.47, t(86) = 3.078, *p* = 0.003, Fig. 5c). This relationship was fully mediated by perceived accuracy (Index of indirect effect = 0.12, SD = 0.37, t(92) = 3.070, p = 0.003). Specifically, repetition was related to higher perceived accuracy (β = 0.23, SD =0.57, t(92) = 3.820, p < 0.001) which in turn was associated with greater sharing (β = 0.46, SD =0.36, t(92) = 12.393, p < 0.001). Once again, after accounting for perceived accuracy the relation between repetition and sharing was not significant (β = 0.16, SD = 1.47, t(92) = 1.056, *p* = 0.294).

## 4 Discussion

Here, we provide evidence for a mechanism which facilitates the spread of misinformation. In particular, we demonstrate that the well-known ‘illusory truth effect’ fuels the spread of misinformation. It has been suggested that a single exposure to repeated information boosts its accuracy perception (for a review Dechêne et al., 2010) – here we show that by doing so it also boosts the spread of said information.

Specifically, our data reveal that people are more likely to share information they have been previously exposed to. We show that the relationship between repetition and sharing is mediated by perceived accuracy. That is, repeated information seems to be shared more because people judge repeated information as more accurate. Our results help explain why fake news spread so easily among the population. Fake-new is often constructed to be appealing to the reader and consequently is more likely to be repeated by different sources (Vosoughi, Roy, & Aral, 2018). Results of our study suggest that repeated exposure to misinformation will create a vicious circle in which misinformation will be perceived as true and therefore shared more. These results stress the importance of quickly tagging misinformation as such. If repeated exposure biases people to share news more, the longer information circulates, the higher the probability that it will be considered as true and further shared with others.

Importantly, repetition increased sharing intentions in different domains. We show that the results replicate for health-related information as well as ‘general knowledge’, suggesting that the effect of repetition on information-sharing generalize beyond the health domain. Future studies could test whether the effect is also present in the political domain, which this study did not do.

In sum, we show that even a single previous exposure to information will increase the likelihood of sharing by enhancing perceived accuracy. This will create a viscous cycle of exposure – increase belief – sharing – exposure - increase belief - which in turn can influence actions. For example, misinformation about COVID-19 vaccines can increases vaccine hesitancy and as a result reduce the likelihood of vaccine uptake.

## Data and code availability

Anonymized data and code are available at a dedicated Github repository https://github.com/affective-brain-lab/The-illusory-truth-effect-leads-to-the-spread-of-misinformation.git.

## CRediT authorship contribution statement

**Valentina Vellani:** Conceptualization, Methodology, Formal analysis, Investigation, Resources, Data curation, Writing – original draft, Visualization. **Sarah Zheng:** Conceptualization, Methodology, Writing – review & editing. **Dilay Ercelik:** Investigation, Resources. **Tali Sharot:** Funding acquisition, Conceptualization, Methodology, Formal analysis, Writing – original draft.

## Data Availability

Data and script are on GitHub, link is provided

## Appendix A Supplementary data

The Supplementary Materials contain a list of stimuli, task instructions, and a supplementary analysis investigating whether social media use is associated with the main findings. Image 1
