# Supplementary material for: The illusory truth effect leads to the spread of misinformation
Source: Cognition. 2023 Jul;236:105421. doi: 10.1016/j.cognition.2023.105421 (PMC10636596; doi:10.1016/j.cognition.2023.105421)
Supplement: Supplementary file 1 — The Supplementary Materials contain a list of stimuli, task instructions, and a supplementary analysis investigating whether social media use is associated with the main findings. [file mmc1.docx]

**Supplementary Materials**

**Stimuli, Exp 1**

| **Statements** | **Source** |
| --- | --- |
| 1.     Sugar-sweetened beverages decrease risk of heart disease | https://www.healthline.com/nutrition/27-health-and-nutrition-tips#TOC_TITLE_HDR_2 |
| 2.     Adults should eat no more than 6g of salt a day | <https://www.nhs.uk/live-well/eat-well/salt-nutrition/> |
| 3.     Art can improve your enjoyment of life and your health | <https://www.mentalhealth.org.uk/blog/how-arts-can-help-improve-your-mental-health> |
| 4.     Avocados are different than most fruits because they are loaded with healthy fats instead of carbs | <https://www.healthline.com/nutrition/avocados-and-weight> |
| 5.     Caffeine consumption reduces bone growth in kids. | https://www.businessinsider.com/worst-science-health-body-myths-2016-8?r=US&IR=T#myth-coffee-stunts-your-growth-8 |
| 6.     Being optimist has strong links with longevity | <https://greatergood.berkeley.edu/article/item/how_optimism_may_keep_you_alive_longer> |
| 7.     Bottled water is better for one's health than tap water | <https://www.castlewater.co.uk/blog/is-bottled-water-healthier-than-tap-water> |
| 8.     Spicy food helps weight loss by encouraging thermogenesis, the process of creating heat from burning fat | <https://www.healthshots.com/healthy-eating/nutrition/eating-spicy-foods-can-help-you-lose-weight-here-is-how/> |
| 9.     Eating a lot of carrots gives you great night vision. | https://www.businessinsider.com/worst-science-health-body-myths-2016-8?r=US&IR=T#myth-coffee-stunts-your-growth-8 |
| 10.  Drinking hot drinks is more effective for cooling off than drinking cold drinks | <https://www.mirror.co.uk/science/drinking-hot-tea-summer-really-18668799> |
| 11.  Drinking more alcohol can cure an ongoing hangover | <https://my.clevelandclinic.org/health/diseases/16627-hangover> |
| 12.  Eating at night may cause trouble sleeping | <https://www.conehealth.com/services/sleep-disorders/late-night-snacks-and-better-sleep-how-what-and-when-you-eat-imp/> |
| 13.  Humans can't grow new brain cells. | https://www.businessinsider.com/worst-science-health-body-myths-2016-8?r=US&IR=T#myth-coffee-stunts-your-growth-8 |
| 14.  Eating too many carrots can lead to a condition called carotenemia which is an orange skin discoloration | <https://uamshealth.com/medical-myths/if-you-eat-too-many-carrots-will-your-skin-turn-orange/> |
| 15.  Electronic screens emit blue light, which suppresses the sleep-inducing hormone melatonin | <https://www.sleep.org/ways-technology-affects-sleep/> |
| 16.  Everyone needs about 8 hours of sleep every day | <https://qz.com/quartzy/1476218/how-much-sleep-do-i-need-the-eight-hour-rule-is-a-myth/> |
| 17.  For better health, one needs to remove sugar entirely from one's diet | <https://www.medicalnewstoday.com/articles/288088> |
| 18.  Garlic can relieve toothaches by releasing allicin | <https://www.healthline.com/health/garlic-for-toothache> |
| 19.  Ginger has potent anti-inflammatory and antioxidant effects | <https://www.healthline.com/nutrition/11-proven-benefits-of-ginger> |
| 20.  Maintaining good relationships can reduce harmful levels of stress | <https://www.betterhealth.vic.gov.au/health/healthyliving/Strong-relationships-strong-health> |
| 21.  It takes 7 years for gum to digest if you swallow it. | https://www.businessinsider.com/worst-science-health-body-myths-2016-8?r=US&IR=T#myth-coffee-stunts-your-growth-8 |
| 22.  You lose 90% of your body heat through your head. | <https://www.businessinsider.com/worst-science-health-body-myths-2016-8?r=US&IR=T#myth-coffee-stunts-your-growth-8> |
| 23.  People who have close friends and family are healthier and live much longer | <https://www.health.harvard.edu/healthbeat/strengthen-relationships-for-longer-healthier-life> |
| 24.  Bananas are among the world’s best sources of potassium | <https://readingpartners.org/blog/four-compelling-reasons-shut-off-screen-open-good-book/> |
| 25.  Speaking positive affirmations out loud can boost self-esteem and keep us motivated | <https://positivepsychology.com/daily-affirmations/> |
| 26.  Sugary drinks are among the most fattening items you can put into your body | <https://www.hsph.harvard.edu/nutritionsource/healthy-drinks/sugary-drinks/> |
| 27.  To avoid cramps and drowning, one needs to wait an hour after eating to swim | <https://www.dignityhealth.org/articles/is-swimming-after-eating-really-dangerous> |
| 28.  Washing your hands is an excellent way to stave off infection and food poisoning | <https://www.nidirect.gov.uk/articles/hand-washing-and-hand-hygiene> |
| 29.  Being cold can give you a cold. | https://www.businessinsider.com/worst-science-health-body-myths-2016-8?r=US&IR=T#myth-coffee-stunts-your-growth-8 |
| 30.  Going out with wet hair gets you sick | https://health.clevelandclinic.org/can-wet-hair-make-you-sick/#:~:text=You%20cannot%20get%20sick%20from,involved%20to%20cause%20a%20cold.%E2%80%9D |
| 31.  Eating food within five seconds of dropping it on the floor is safe | https://www.cnet.com/health/common-health-myths-you-need-to-stop-believing-right-now/ |
| 32.  Bananas are suggested for a low carbs diet | <https://www.healthline.com/nutrition/14-foods-to-avoid-on-low-carb> |
| 33.  Microwave ovens is bad for your health | https://www.healthline.com/nutrition/microwave-ovens-and-health#harmful-compounds |
| 34.  Canned foods have little nutritional value | <https://www.healthline.com/nutrition/canned-food-good-or-bad> |
| 35.  Gluten should be removed from our diet | https://www.coeliac.org.uk/information-and-support/living-gluten-free/the-gluten-free-diet/?&&type=rfst&set=true#cookie-widget |
| 36.  Chocolate is an aphrodisiac | <https://theconversation.com/mondays-medical-myth-chocolate-is-an-aphrodisiac-4980> |
| 37.  Crusts are the most nutritious part of the bread in terms of the quantity of antioxidants | <https://edition.cnn.com/2018/03/27/health/bread-crust-food-drayer/index.html> |
| 38.  Cracking joints causes arthritis | https://www.webmd.com/balance/ss/slideshow-10-health-myths-debunked |
| 39.  Eating sugar is associated to poor focus in kids | https://www.webmd.com/balance/ss/slideshow-10-health-myths-debunked |
| 40.  During the weekend, you can catch up on sleep you have missed during the weekdays, preventing negative health outcomes | <https://www.sleepfoundation.org/how-sleep-works/sleep-debt-and-catch-up-sleep> |
| 41.  Eating enough protein is particularly important for weight loss and for maintaining good health overall | <https://www.healthline.com/nutrition/how-protein-can-help-you-lose-weight> |
| 42.  Eating fiber-based food is recommended for maintaining good gut health | <https://www.medicalnewstoday.com/articles/short-term-increase-in-fiber-alters-gut-microbiome> |
| 43.  Eating slowly gives your brain the chance to get the signal that you’re full | <https://www.health.harvard.edu/blog/why-eating-slowly-may-help-you-feel-full-faster-20101019605> |
| 44.  Eating sugar is the direct cause of diabetes in the population | <https://www.medicalnewstoday.com/articles/317246> |
| 45.  Eating yogurt helps put the digestive system back in order thanks to its probiotics | <https://www.npr.org/2011/10/28/141800414/does-probiotic-yogurt-really-affect-digestion> |
| 46.  Eggs increase the risk for heart disease and atherosclerosis | https://www.health.harvard.edu/heart-health/are-eggs-risky-for-heart-health |
| 47.  Fatty fish is extremely beneficial to health due to the concentration of omega-3 fatty acids | <https://www.mayoclinic.org/diseases-conditions/heart-disease/in-depth/omega-3/art-20045614> |
| 48.  Juice cleanses enhances the body's ability to cleanse itself | [https://www.mdanderson.org/publications/focused-on-health/FOH-cleanses-detox-fasts.h10-1590624.html)](https://www.verywellfit.com/juice-cleanse-89120) |
| 49.  Nuts and seed are very low in proteins and fibers | https://www.healthline.com/nutrition/27-health-and-nutrition-tips#TOC_TITLE_HDR_2 |
| 50.  Laughing is good for the heart and can increase blood flow by 20% | <https://www.scientificamerican.com/article/laughter-proves-good-medi/> |
| 51.  Mindful breathing can keep you present and help you centre your attention | <https://ggia.berkeley.edu/practice/mindful_breathing> |
| 52.  People who consume extra virgin olive oil have a much lower risk of dying from heart attacks | <https://www.bhf.org.uk/what-we-do/news-from-the-bhf/news-archive/2014/may/olive-oil-benefits> |
| 53.  Pickle juice alleviates muscle cramps | https://www.healthline.com/health/pickle-juice-for-cramps |
| 54.  Poor sleep can reduce your physical and mental performance | <https://www.webmd.com/sleep-disorders/features/emotions-cognitive> |
| 55.  Refined carbs consumption is discouraged because of its low concentration in fiber | <https://www.healthline.com/nutrition/why-refined-carbs-are-bad> |
| 56.  Sunlight is a source of vitamin D, which helps our brains release mood-boosting endorphins and serotonin | https://www.healthline.com/health/depression/benefits- https://www.bbc.co.uk/programmes/articles/22DdHRHcn64x1l4yZ6hygMc/why-you-should-be-catching-the-last-rays-of-summer-sun |
| 57.  Tomatoes are usually categorized as a vegetable, although they are technically a fruit | <https://www.eufic.org/en/healthy-living/article/is-a-tomato-a-fruit-or-a-vegetable-and-why> |
| 58.  Using deodorant with aluminium-based compounds can cause diseases | <https://www.webmd.com/breast-cancer/features/antiperspirant-facts-safety> |
| 59.  Walking for 20-30 minutes a day, five days a week can improve your immune system | <https://www.health.harvard.edu/staying-healthy/5-surprising-benefits-of-walking> |
| 60.  Yoga helps bone health and boosts the immune system | <https://www.active.com/fitness/articles/how-yoga-can-help-boost-your-immune-system> |

**Stimuli, Exp 2**

| **Statement** | **Topic** | **Source** |
| --- | --- | --- |
| 1.The thigh bone is the largest bone in the human body | Science | [Arkes et al. (1989)](https://onlinelibrary.wiley.com/doi/epdf/10.1002/bdm.3960020203?saml_referrer) |
| 2.Mexico is the world’s largest producer of silver | Geography | [Arkes et al. (1989)](https://onlinelibrary.wiley.com/doi/epdf/10.1002/bdm.3960020203?saml_referrer) |
| 3.The largest dam in the world is in Pakistan | Geography | [Arkes et al. (1989)](https://onlinelibrary.wiley.com/doi/epdf/10.1002/bdm.3960020203?saml_referrer) |
| 4.The Cyclops is the legendary one-eyed giant in Greek mythology | Literature | [Fazio et al. (2015)](https://psycnet.apa.org/manuscript/2015-38275-001.pdf) |
| 5.Marconi is the inventor of the wireless radio | Science | [Fazio et al. (2015)](https://psycnet.apa.org/manuscript/2015-38275-001.pdf) |
| 6.The largest planet in the solar system is Jupiter | Science | [Brashier et al. (2020)](https://www.sciencedirect.com/science/article/pii/S0010027719302276#s0180) |
| 7.Volleyball was originally called mintonette | Sport | [Newman et al. (2020)](https://www.sciencedirect.com/science/article/pii/S1053810019301977?via%3Dihub#m0020) |
| 8.Walruses use their tusks primarily for mating | Science | [Newman et al. (2020)](https://www.sciencedirect.com/science/article/pii/S1053810019301977?via%3Dihub#m0020) |
| 9.Greenland is a part of the Kingdom of Denmark | Geography | [Newman et al. (2020)](https://www.sciencedirect.com/science/article/pii/S1053810019301977?via%3Dihub#m0020) |
| 10.The stationary ball in lawn bowls is called a jack | Sport | [Newman et al. (2020)](https://www.sciencedirect.com/science/article/pii/S1053810019301977?via%3Dihub#m0020) |
| 11.Domesticated goats are descended from the pasang | Science | [Newman et al. (2020)](https://www.sciencedirect.com/science/article/pii/S1053810019301977?via%3Dihub#m0020) |
| 12.Kava is a beverage made from the root of the pepper plant | Food | [Newman et al. (2020)](https://www.sciencedirect.com/science/article/pii/S1053810019301977?via%3Dihub#m0020) |
| 13.Lake Baikal is the world's largest freshwater lake by volume | Geography | [Newman et al. (2020)](https://www.sciencedirect.com/science/article/pii/S1053810019301977?via%3Dihub#m0020) |
| 14.Female turkeys generally weigh half as much as males | Science | [Newman et al. (2020)](https://www.sciencedirect.com/science/article/pii/S1053810019301977?via%3Dihub#m0020) |
| 15.The lima bean is also known as the sieva bean | Food | [Newman et al. (2020)](https://www.sciencedirect.com/science/article/pii/S1053810019301977?via%3Dihub#m0020) |
| 16.Halvah is a confection made of sesame seeds | Food | [Newman et al. (2020)](https://www.sciencedirect.com/science/article/pii/S1053810019301977?via%3Dihub#m0020) |
| 17.Normal color vision is known as trichromacy | Science | [Newman et al. (2020)](https://www.sciencedirect.com/science/article/pii/S1053810019301977?via%3Dihub#m0020) |
| 18.The stones used in curling are concave on the bottom | Sport | [Newman et al. (2020)](https://www.sciencedirect.com/science/article/pii/S1053810019301977?via%3Dihub#m0020) |
| 19.The tool that plots position relative to the poles is a compass | Science | [Brashier et al. (2020)](https://www.sciencedirect.com/science/article/pii/S0010027719302276#s0180) |
| 20.The ship that carried the Pilgrims to America is the Mayflower | History | [Brashier et al. (2020)](https://www.sciencedirect.com/science/article/pii/S0010027719302276#s0180) |
| 21.The world famous magician and escape artist was Houdini | Culture | [Brashier et al. (2020)](https://www.sciencedirect.com/science/article/pii/S0010027719302276#s0180) |
| 22.Molten rock that runs down the side of a volcano is lava | Science | [Brashier et al. (2020)](https://www.sciencedirect.com/science/article/pii/S0010027719302276#s0180) |
| 23.The men who flew the first airplane were the Wright brothers | Culture | [Brashier et al. (2020)](https://www.sciencedirect.com/science/article/pii/S0010027719302276#s0180) |
| 24.The liquid portion of whole blood is plasma | Science | [Brashier et al. (2020)](https://www.sciencedirect.com/science/article/pii/S0010027719302276#s0180) |
| 25.A rider on horseback hits a ball with his mallet in polo | Sport | [Brashier et al. (2020)](https://www.sciencedirect.com/science/article/pii/S0010027719302276#s0180) |
| 26.Severe headaches accompanied by nausea are migraines | Science | [Brashier et al. (2020)](https://www.sciencedirect.com/science/article/pii/S0010027719302276#s0180) |
| 27.The ocean between Africa and Australia is the Indian Ocean | Geography | [Brashier et al. (2020)](https://www.sciencedirect.com/science/article/pii/S0010027719302276#s0180) |
| 28.The Italian city known for its canals is Venice | Geography | [Brashier et al. (2020)](https://www.sciencedirect.com/science/article/pii/S0010027719302276#s0180) |
| 29.A giant ocean wave caused by an earthquake is a tsunami | Science | [Brashier et al. (2020)](https://www.sciencedirect.com/science/article/pii/S0010027719302276#s0180) |
| 30.The outer layer of cheese is known as the rind | Food | [Brashier et al. (2020)](https://www.sciencedirect.com/science/article/pii/S0010027719302276#s0180) |
| 31.New Delhi, India, is the world’s most populous city | Geography | [Arkes et al. (1989)](https://onlinelibrary.wiley.com/doi/epdf/10.1002/bdm.3960020203?saml_referrer) |
| 32.The capital of Russia is Saint Petersburg | Geography | [Brashier et al. (2020)](https://www.sciencedirect.com/science/article/pii/S0010027719302276#s0180) |
| 33.The Indian Ocean is the smallest ocean on Earth | Geography | [Arkes et al. (1989)](https://onlinelibrary.wiley.com/doi/epdf/10.1002/bdm.3960020203?saml_referrer) |
| 34.The planet Venus is larger than the planet Earth | Science | [Arkes et al. (1989)](https://onlinelibrary.wiley.com/doi/epdf/10.1002/bdm.3960020203?saml_referrer) |
| 35.The Atlantic Ocean is the largest ocean on Earth | Geography | [Fazio et al. (2015)](https://psycnet.apa.org/manuscript/2015-38275-001.pdf) |
| 36.Bell is the inventor of the wireless radio | Science | [Fazio et al. (2015)](https://psycnet.apa.org/manuscript/2015-38275-001.pdf) |
| 37.The capital of New York is New York City | Geography | [Brashier et al. (2020)](https://www.sciencedirect.com/science/article/pii/S0010027719302276#s0180) |
| 38.The Chicago Marathon is the world’s oldest annual marathon | Sport | [Newman et al. (2020)](https://www.sciencedirect.com/science/article/pii/S1053810019301977?via%3Dihub#m0020) |
| 39.Kvass is an alcoholic beverage fermented from honey | Food | [Newman et al. (2020)](https://www.sciencedirect.com/science/article/pii/S1053810019301977?via%3Dihub#m0020) |
| 40.The monetary unit in Afghanistan is the rupee | Culture | [Newman et al. (2020)](https://www.sciencedirect.com/science/article/pii/S1053810019301977?via%3Dihub#m0020) |
| 41. Europe has the highest average elevation of the continents | Geography | [Newman et al. (2020)](https://www.sciencedirect.com/science/article/pii/S1053810019301977?via%3Dihub#m0020) |
| 42.Spain produces most of the world's almonds | Food | [Newman et al. (2020)](https://www.sciencedirect.com/science/article/pii/S1053810019301977?via%3Dihub#m0020) |
| 43.Competitive badminton is usually played outdoors | Sport | [Newman et al. (2020)](https://www.sciencedirect.com/science/article/pii/S1053810019301977?via%3Dihub#m0020) |
| 44.Dough is boiled in the process of making croissants | Food | [Newman et al. (2020)](https://www.sciencedirect.com/science/article/pii/S1053810019301977?via%3Dihub#m0020) |
| 45.The highest waterfall in the world is in Argentina | Geography | [Newman et al. (2020)](https://www.sciencedirect.com/science/article/pii/S1053810019301977?via%3Dihub#m0020) |
| 46.Biking is the first event in a triathlon | Sport | [Newman et al. (2020)](https://www.sciencedirect.com/science/article/pii/S1053810019301977?via%3Dihub#m0020) |
| 47.The mouth of a sea urchin is on its top | Science | [Newman et al. (2020)](https://www.sciencedirect.com/science/article/pii/S1053810019301977?via%3Dihub#m0020) |
| 48.Candlepins is the most widely played variation of bowling | Sport | [Newman et al. (2020)](https://www.sciencedirect.com/science/article/pii/S1053810019301977?via%3Dihub#m0020) |
| 49. Tennis has been traced back to the baths of Rome | Sport | [Newman et al. (2020)](https://www.sciencedirect.com/science/article/pii/S1053810019301977?via%3Dihub#m0020) |
| 50.Endothermic reactions release chemical energy | Science | [Newman et al. (2020)](https://www.sciencedirect.com/science/article/pii/S1053810019301977?via%3Dihub#m0020) |
| 51.The thick layer of fat on a whale is its peduncle | Science | [Brashier et al. (2020)](https://www.sciencedirect.com/science/article/pii/S0010027719302276#s0180) |
| 52.Michelangelo painted the ceiling of Saint Peter's Basilica | Culture | [Brashier et al. (2020)](https://www.sciencedirect.com/science/article/pii/S0010027719302276#s0180) |
| 53.Plants make their food during chemosynthesis | Science | [Brashier et al. (2020)](https://www.sciencedirect.com/science/article/pii/S0010027719302276#s0180) |
| 54.Abraham Lincoln was assassinated by Ray | History | [Brashier et al. (2020)](https://www.sciencedirect.com/science/article/pii/S0010027719302276#s0180) |
| 55.The name for the collar bone is the scapula | Science | [Brashier et al. (2020)](https://www.sciencedirect.com/science/article/pii/S0010027719302276#s0180) |
| 56.The sport associated with Wimbledon is field hockey | Sport | [Brashier et al. (2020)](https://www.sciencedirect.com/science/article/pii/S0010027719302276#s0180) |
| 57.The villainous captain in the story 'Peter Pan' is Captain Smee | Literature | [Brashier et al. (2020)](https://www.sciencedirect.com/science/article/pii/S0010027719302276#s0180) |
| 58.The name of Tarzan's girlfriend is Marian | Literature | [Brashier et al. (2020)](https://www.sciencedirect.com/science/article/pii/S0010027719302276#s0180) |
| 59.The short pleated skirt worn by Scottish men is a sari | Culture | [Brashier et al. (2020)](https://www.sciencedirect.com/science/article/pii/S0010027719302276#s0180) |
| 60. Old Faithful is located in Yosemite Park | Geography | [Brashier et al. (2020)](https://www.sciencedirect.com/science/article/pii/S0010027719302276#s0180) |

**Supplementary Analysis and Results**

To examine if social media use impacted the results participants completed questions assessing their social media use. As each social media question had a different scale, we rescaled each question so that it ranged from 0 to 1 (using the R function “rescale”). We then averaged all questions to obtain a single social media score. We added this score to the Linear Mixed Models described in the main text as a fixed effect.  As in the main model, repetition, ground truth and their interactions were modelled as fixed and random effect. Random and fixed intercepts were also included. In both Exp1 and Exp 2, the effect of repetition on sharing intentions were significant (Exp 1: β = -2.08, t(317.35) =-2.52, p = 0.012; Exp 2: β = -0.16, t(267.27) = -2.91, p = 0.004) as was perceived accuracy (Exp 1: β = -3.12, t(315.96) =-3.857, p < 0.001; Exp 2: β = -0.19, t(247.94) = -2.91, p = 0.004). Social media was related to greater sharing behavior (β = 18.05, t(157.98) = 2.52, p = 0.026) and perceived accuracy (β = 7.25, t(157.97) = 2.03, p = 0.044) in Exp1, but not in Exp2 (Sharing: β = 0.64, t(98) = 1.33, p = 0.18; Perceived Accuracy: β = 0.15, t(97.99) = 0.70, p = 0.48).

In order to account for any possible effect of item identity we performed a Linear Mixed Model predicting sharing behaviour with item as a random effect. Fixed effects included repetition and ground truth and their interaction. Random effects included repetition (as accuracy is fixed per item). Fixed and random intercepts were also modelled. Results confirmed sharing was greater for repeated items than new items (Exp 1: β = -2.1, t(57.81) =-2.39, p = 0.02; Exp 2: β = -0.17, t(58.09) = -2.73, p = 0.008) and for true items than misinformation (Exp 1: β = -26.41, t(65.04) =-10.102, p < 0.001; Exp 2: β = -0.69, t(71.848) = -5.008, p < 0.001). No other effects were significant.

For Exp 2, the same model with item as a random effect was run after removing four statements which are likely easier to be asses by a US sample (item number: 37, 38, 54, 60), to test if this will alter the results in our mostly British sample. Results once again confirmed that participants shared repeated information more than new information (β = -0.17, t(54.08) =-2.66, p = 0.01) and true information more than missinformation (β = -0.71, t(66.99) =-4.86, p < 0.001). No other effects were significant.

**Task Instructions in Block 1**

**Exp1**

In this task you will decide which information related to health and life-style to share on a Twitter page that will be shown to participants who will complete a similar task tomorrow.

Note, that during the task we could also ask you to report the information you have just seen

or we could ask you questions about the information you have just seen. So, pay full attention to the information presented

**Exp2**

In this task you will manage a Twitter page about general knowledge.

Your job is to decide which information to share on your Twitter page.

In the first part of the task on each trial we will present you with a statement.

Note, that during the task we could also ask you to report the information you have just seen or we could ask you questions about the information you have just seen. So, pay full attention to the information presented
